# The differential modulation of secondary metabolism induced by a protein hydrolysate and a seaweed extract in tomato plants under salinity

**DOI:** 10.3389/fpls.2022.1072782

**Published:** 2023-01-16

**Authors:** Leilei Zhang, Giorgio Freschi, Youssef Rouphael, Stefania De Pascale, Luigi Lucini

**Affiliations:** ^1^ Department for Sustainable Food Process, Università Cattolica del Sacro Cuore, Piacenza, Italy; ^2^ Agro Unit, Clever Bioscence srl, Pavia, Italy; ^3^ Department of Agricultural Sciences, University of Naples Federico II, Portici, Italy

**Keywords:** biostimulants, metabolomics, plant stress, secondary metabolism, phytohormones

## Abstract

Climate change and abiotic stress challenges in crops are threatening world food production. Among others, salinity affects the agricultural sector by significantly impacting yield losses. Plant biostimulants have received increasing attention in the agricultural industry due to their ability to improve health and resilience in crops. The main driving force of these products lies in their ability to modulate plant metabolic processes involved in the stress response. This study’s purpose was to investigate the effect of two biostimulant products, including a protein hydrolysate (Clever HX^®^) and a seaweed extract with high amino acids content (Ascovip^®^), and their combination, on the metabolomics profile of tomato crops grown under salt stress (150 mM NaCl). Several stress indicators (leaf relative water content, membrane stability index, and photosynthesis activity) and leaf mineral composition after salinity stress exposure were assessed to evaluate stress mitigation, together with growth parameters (shoot and root biomasses). After that, an untargeted metabolomics approach was used to investigate the mechanism of action of the biostimulants and their link with the increased resilience to stress. The application of the biostimulants used reduced the detrimental effect of salinity. In saline conditions, protein hydrolysate improved shoot dry weight while seaweed extracts improved root dry weight. Regarding stress indicators, the application of the protein hydrolysate was found to alleviate the membrane damage caused by salinity stress compared to untreated plants. Surprisingly, photosynthetic activity significantly improved after treatment with seaweed extracts, suggesting a close correlation between root development, root water assimilation capacity and photosynthetic activity. Considering the metabolic reprogramming after plant biostimulants application, protein hydrolysates and their combination with seaweed extracts reported a distinctive metabolic profile modulation, mainly in secondary metabolite, lipids and fatty acids, and phytohormones biosynthetic pathways. However, treatment with seaweed extract reported a similar metabolic reprogramming trend compared to salinity stress. Our findings indicate a different mechanism of action modulated by protein hydrolysate and seaweed extract, suggesting stronger activity as a stress mitigator of protein hydrolysate in tomato crops under salinity stress.

## 1 Introduction

Among the crop abiotic stresses that threaten world food security, soil salinity is considered a major challenge that affects the global agriculture sector, causing significant losses in production each year. Nowadays, about 20% of irrigated lands (1500 million hectares) are damaged by high salt content, and this percentage is estimated to grow to 50% (3750 million hectares) by the end of 2050 (Jamil et al., 2011; Chung et al., 2020; Kumar et al., 2020). The main reason for the increasing soil salinity accumulation lies in using saline water for irrigation, poor water management, high evaporation, and previous exposure to seawater. Soil salinity implies the presence of any salt at higher levels, including sodium ions (Na^+^), chloride (Cl^–^), sulfurates ( ${\text{SO}}_{4}^{2-}$ ), nitrates ( ${\text{NO}}_{3}^{-}$ ), borates ( ${\text{BO}}_{3}^{3-}$ ), carbonates ( ${\text{CO}}_{3}^{2-}$ ), bicarbonates ( ${\text{HCO}}_{3}^{-}$ ), calcium (Ca^2+^), magnesium (Mg^2+^), potassium (K^+^), and ion (Fe^2+^), which can exert adverse effects on plants (Bui, 2017). Indeed, salinity is well documented to affect the agronomical, physiological, and biochemical processes of plants (EL Sabagh et al., 2020; Khan et al., 2021). At the agronomical level, salinity stress causes a loss of both shoot and root biomass development and a reduction in crop productivity (EL Sabagh et al., 2020). While at the physiological level, it is able to cause ionic imbalances and ion toxicity through competition with several essential minerals such as K^+^, Mg^2+^, or Ca^2+^ ions, thus leading to chlorosis and necrosis of leaves (Almeida et al., 2017). Finally, salinity stress also induces biochemical changes in plants, including phytohormones modulation, ion uptake alteration, antioxidant enzyme activation, reactive oxygen species (ROS) accumulation, and photosynthetic pathway disruption and consequently compromising chlorophyll and carotenoids content and photosystem II (PSII) activity (Yoon et al., 2009; Chung et al., 2020).

Different strategies have been adopted to cope with this important issue, including traditional breeding, genetic engineering, and chemical fertilizer applications, which are not always feasible and sustainable for the ecosystem (Kumar et al., 2020). Thus, there is an urgent need to adopt and develop new innovative and eco-friendly strategies to manage this challenge in the agricultural systems. In the last decades, the use of plant biostimulants gained enormous importance as plant growth promoting products and plant defence elicitors, and is the most promising solution to cope with salinity stress (Rouphael et al., 2020). The latest European regulation on fertilizers, which includes biostimulants (REGULATION (EU) 2019/1009 OF, 2019), gave the latest definition of biostimulants: “Biostimulants are organic or inorganic products containing bioactive substances and/or microorganisms, which, when applied to the plant or rhizosphere, stimulate the growth and productivity of the plant by improving the absorption and assimilation efficiency of nutrients, tolerance to abiotic stresses and/or quality of the product regardless of their nutrient content”.

Based on the definition above, there are two types of biostimulants: microbial biostimulants and non-microbial biostimulants. Focusing on non-microbial biostimulants, which are usually distinguished based on the starting material used to produce them, the most important ones are protein hydrolysate (PH) and seaweed extracts (SW). PHs are obtained by biological matrices after an optimised hydrolysis process that includes either chemical or enzymatic hydrolysis. PHs are mainly characterised by a mixture of amino acids, peptides, and proteins (Colla et al., 2015). Whereas SWs are produced by macroscopic marine algae belonging to different taxonomic groups, such as brown, red, and green algae (Khan et al., 2009). SWs are rich sources of nutrients, bioactive compounds, phytohormones, minerals, and organic matters, as well as characterised by complex polysaccharides such as laminarin, fucoidan, and alginates (Abraham et al., 2019; Flórez-Fernández et al., 2019). In the case of plants affected by salinity stress, the biostimulants were usually employed to overcome the osmotic and ionic stress by modulating both primary and secondary metabolism (Rouphael et al., 2020), and improving crops’ growth, productivity, and tolerance to abiotic stresses (Rouphael et al., 2017; Paul et al., 2019a).

Despite the positive effect of biostimulants application in coping with various abiotic stresses, the crucial point highlighted by the latest European legislation on fertilizers includes the presence of scientific evidence to support the efficacy of a product registered as a biostimulant, including detailed clarification of their mechanism of action (REGULATION (EU) 2019/1009 OF, 2019). In recent years, the omics sciences (genomics, transcriptomics, proteomics and metabolomics) have gained a lot of notoriety in addressing this issue (Sahoo et al., 2020). Among these, metabolomics has contributed the most to understanding the mechanisms of action of plant biostimulants against abiotic stress (Paul et al., 2019a; Paul et al., 2019b; Nephali et al., 2020). Specifically, metabolomics is a qualitative and quantitative study of all metabolites involved in metabolic processes responsible for the maintenance of the normal function of an organism. In particular, the untargeted metabolomics approach has shown to have a high potential for unravelling the biochemical processes of plants affected by stresses and those regulated after biostimulant application (Burgess et al., 2014; Schrimpe-Rutledge et al., 2016; Rouphael et al., 2020).

This work aims to investigate and compare the potential salt stress mitigation effect of two biostimulant products such as PH (Clever HX^®^), SW (Ascovip^®^), and their combination PH + SW, applied by foliar spray on tomato plant (*Solanum lycopersicum* L.). For this purpose, agronomical, physiological, and biochemical parameters were assessed after salinity stress exposure and compared with the corresponding treatment with biostimulants’ application. To this aim, an untargeted metabolomics analysis was used to comprehensively investigate the mechanism of action of the biostimulants and to unravel the processes underlying their stress tolerance mitigation. Considering that plant biostimulants can provide an extensive modulation of crop metabolism (Colla et al., 2015), and given the limited knowledge on the products used in this study, metabolomics has been chosen because of its hypothesis-free untargeted nature to better achieve the goal of the study.

## 2 Materials and methods

### 2.1 Plant material and growth conditions

Tomato plants (*Solanum lycopersicum* L., cv. Heinz 3402) were provided by a local nursery (Azienda F.lli Zermani, Piacenza, Italy) and transplanted in single squared pots (12 cm x 12cm x 14 cm) at the four true leaves stage. After that, plants were grown for 18 days, starting from 21^st^ of June 2021 to 8^th^ of July 2021, under natural open field conditions at the experimental station of Università Cattolica del Sacro Cuore (Piacenza, Emilia-Romagna, Italy). The soil used for the experiment was a commercial Radicom universal potting soil contains a mix of fine white and brown peat (65%), green compost, Ecofibra^®^, and coir fibre (Vigorplant Italia srl, Fombio, LO, Italy). The specific characteristic of soil includes pH = 7.5, 0.4 ds/m electrical conductance, 180 kg/m^3^ density, and 87% *v/v* total porosity.

### 2.2 Biostimulants

Two commercial biostimulants were supplied and produced by Clever Bioscience srl (Casanova Lonati PV, Italy). Clever HX^®^ product is a protein hydrolysate characterized by 20% of peptides and 31% of free amino acids. Moreover, it contains 3.2% of organic nitrogen, 3.2% of potassium oxide, and 10% of organic carbon. The aminogram of the product (as %) is as follows: Ala (5.26), Arg (1.52), Asp (3.80), Cys (0.04), Glu (11.29), Gly (2.98), His (0.96), Ile + Leu (2.27), Lys (2.14), Met (0.9), Phe (1.42), Pro (4.23), Ser (2.19), Thr (2.24), Trp (0.34), Tyr (1.71), and Val (2.29). Ascovip^®^ is a seaweed extract characterized by 15% free amino acids, 10% low molecular weight peptones, 10% organic carbon, 2% organic nitrogen, and 1% of betaines.

### 2.3 Experimental design

Tomato plants were randomly distributed into five experimental groups with eight biological replicates per group, amounting to 40 pots. The experimental groups were defined as follows: a control non-stressed (C), 150 mM of salinity stress (S), 150 mM salinity stress treated with Clever HX^®^ 10% (*v/v*; PH), Ascovip^®^ 10% (*v/v*; SW), and their combination 5% + 5% (*v/v*; PH + SW). The biostimulant treatments were performed three times: at 3 days after transplanting (DAT) referred to as Treatment 1 (T1), at 7 DAT referred to as Treatment 2 (T2), and at 14 DAT referred to as Treatment 3 (T3).

The 150 mM sodium chloride solution (Merck KGaA, Darmstadt, Germany), prepared using demineralized water, was applied by irrigation every day to induce salinity stress. The control pots were irrigated using tap water at the same volume (pH= 7.2, conductibility 572 μS/cm). Considering the biostimulants, 2 mL of each product, properly diluted in demineralized water to the concentration indicated above, was applied by foliar spray to each plant. At the end of the experiment, three out of eight replicates were destinated to morphological analysis, while the remaining five plants have been allocated for leaf membrane stability index (MSI), relative water content (RWC), metabolomics analysis, and minerals quantification analysis. Specifically, shoot and root of the three plants destinated for morphological measurements were collected to evaluate plant biomass. Shoot fresh weight (FW) was recorded immediately after sampling, while root samples were recorded after being washed under tap water and dried with paper. Shoots and roots were then dried for 48 hours at 80°C to measure dry weight (DW). Concerning the remaining five plants, one leaf of each plant per treatment was collected for RWC, while two leaves were collected for MSI. The remaining leaves were deep frozen in liquid nitrogen and immediately stored for metabolomics analysis and minerals quantification. For minerals quantification, leaves sample were dried for 48 hours at 80°C.

### 2.4 Leaf relative water content, membranes stability index, chlorophyll content, and photosynthetic activity

For the determination of RWC, one leaf of each of the five replicates was collected per treatment. The leaves were weighted (FW) and then incubated in 15 ml of ultrapure water for 24h at 4°C. After incubation, leaves were blotted and weighted to get the turgid weight (TW). Finally, leaf samples were oven-dried and newly weighted (DW). The RWC was finally determined using formula below indicated


$$RWC=\frac{FW-DW}{TW-DW}\;\times \;100$$

Leaf membrane stability index (MSI) was measured using two leaves from each of the five replicates for treatment. Tomato leaves were transferred in tubes with 5 ml of ultrapure water. They were heated at 30°C for 30 min and electrical conductivity was measured (C1). Samples were then heated in a bath at 100°C for 30 minutes, then cooled on ice, and the electric conductivity was measured again (C2). The MSI index was calculated using formula below indicated.


$$MSI=\left(1-\frac{C1}{C2}\right)\times \;100$$

The effective photochemical quantum yield of photosystem II (Phi2), ratio of incoming light that is lost *via* non-regulated processes (PhiNO), non-photochemical quenching (PhiNPQ), and chlorophyll content were measured at 18 DAT by using a PhotosynQ instrument (PHOTOSYNQ INC., East Lansing, MI, USA).

### 2.5 Mineral and organic acid determination

The dried tomato leaves were extracted in ultrapure water in a 1:5 ratio using a shaking water bath at 80°C for 10 min. The samples were analysed after centrifugation at 10,000 *x g* for 10 min and filtration through a 0.20 µm cellulose cartridge. The quantification of anions ( ${\text{NO}}_{3}^{-}$ , ${\text{PO}}_{4}^{3-}$ , ${\text{SO}}_{4}^{2-}$ , and Cl^-^) and cations (K^+^, Ca^2+^, Mg^2+^, and Na^+^), as well as organic acids (malate, oxalate, citrate, and isocitrate), were determined by using ion chromatography coupled to an electrical conductivity detector (ICS3000, Thermo Scientific™ Dionex™, Sunnyvale, CA, USA) as previously reported (Formisano et al., 2021). Specifically, cations were separated by isocratic chromatography through an IonPac^®^ CS12A column (4 × 250 mm, Thermo Scientific™ Dionex™) with 25 mM methanesulfonic acid (Sigma Aldrich, Milan, Italy). Anions and organic acids were separated by a 5 mM–30 mM potassium hydroxide (KOH) gradient using an IonPac^®^ AG11-HC IC column (4 × 50 mm; Thermo Scientific™ Dionex™) with 5 mM – 30 mM potassium hydroxide (KOH), setting the flow at 1.5 mL min^−1^.

### 2.6 Metabolomics analysis

For each replicate per experimental group, leaf samples were ground in liquid nitrogen with mortar and pestle. Then an aliquot (1 g) was extracted in a methanolic solution (80% methanol + 0.1% formic acid) by homogenization process (Polytron PT 1200 E, Kinematica AG, Switzerland). The homogenised extracts were centrifuged at 5000 × g for 15 minutes and filtered (0.22 µm membrane) into vials ready to be analysed. The untargeted metabolomics analysis was carried out using ultra-high performance liquid chromatography coupled to quadrupole time-of-flight mass spectrometry (UHPLC-QTOF/MS; Agilent Technologies, Santa Clara, CA, USA) as previously described (Pretali et al., 2016). The separation was performed by using an Agilent Poroshell 120 pentafluorophenyl (PFP) column (2.1 × 100 mm, 1.9 μm) and a binary mixture of water and acetonitrile acidified with 0.1% (*v/v*) formic acid as mobile phase (LC-MS grade, VWR, Milan, Italy). A linear gradient (5 to 95% of organic phase) and a 33 min run time were used for separation. The mass spectrometer worked in SCAN mode with a range of 100 to 1200 m/z, positive polarity and extended dynamic range mode, with a nominal mass resolution of 30,000 FWHM. The raw mass features acquired by the instrument were aligned and filtered, then annotated according to the ‘find-by-formula’ algorithm against the PlantCyc database (Hawkins et al., 2021) using the Agilent Profinder B.10.0 software (Agilent Technologies, Santa Clara, CA, USA). To this aim, only the features being putatively annotated within 75% of replications within at least one experimental group were retained, as previously described (Pretali et al., 2016). The annotation follows a LEVEL 2 of identification, referring to COSMOS standards in metabolomics (Salek et al., 2015).

### 2.7 Statistical analysis

The significance of the impact of the treatments on morphological and physiological parameters was investigated with a one-way analysis of the variance (ANOVA), followed by Tukey’s HSD (honestly significant difference) (p-value< 0.05) by using the software PASW Statistics 26.0 (SPSS Inc., Chicago, IL, USA). The chemometrics analysis was carried out using Agilent Mass Profiler Professional B.15.1 (Agilent Technologies, Santa Clara, CA, USA) as previously described (Lucini et al., 2019). Differential compounds were identified by Volcano Plot analysis: the statistically significant compounds (p-value< 0.05, Bonferroni multiple testing correction) having a Fold Change value ≥ 2.5 were selected and used for pathway analysis using Omic Viewer Pathway Tool of PlantCyc (Caspi et al., 2013) (Stanford, CA, USA), to identify the biochemical processes most affected by treatments.

## 3 Results

### 3.1 Foliar biostimulants mitigate the negative effect of salinity stress in tomato plants at agronomical and physiological levels

**Figure 1:**
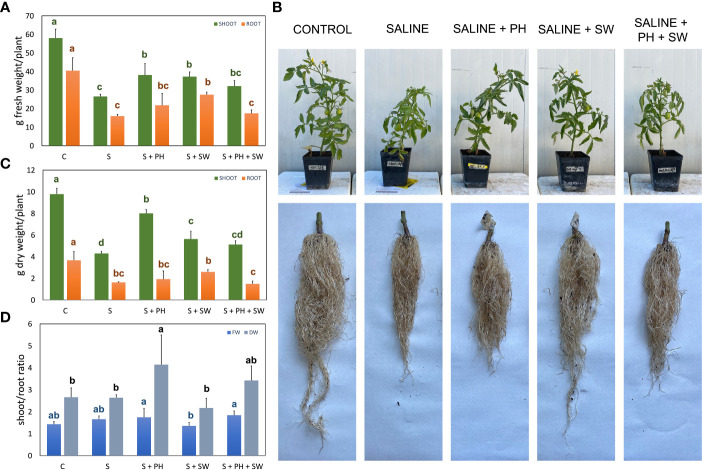
Morphological measurements of tomato shoot and root organ parts, affected by salinity stress **(S)** and treated with biostimulants i.e., protein hydrolysate (PH), seaweed extracts (SW), and their combination PH + SW, compared to the control non-stressed plants **(C)**. In the panels they show **(A)** fresh weight, **(B)** dry weight, and **(C)** the shoot/root ratio of fresh and dry weights for both tomato shoot and roots. In **(D)** shows the image of the aerial parts and roots after cleaning. Error bars indicate standard errors (*n* = 3). Different letters indicate significant differences according to Tukey’s multiple-range test (p = 0.05).

**Figure 2:**
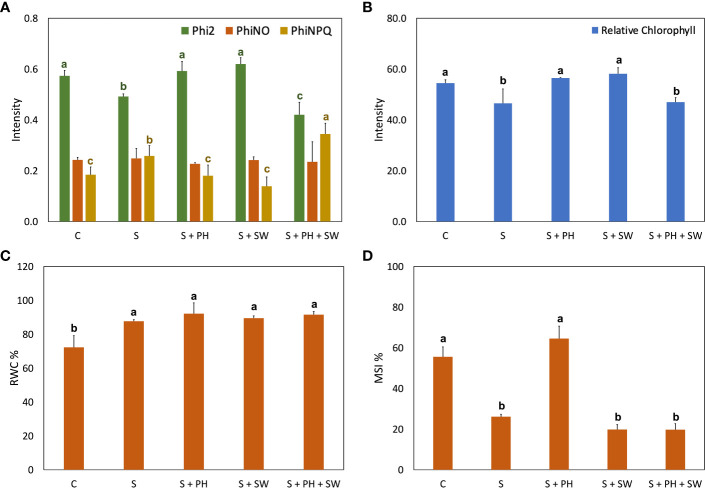
Physiological analysis of tomato leaves affected by salinity stress **(S)** and treated with biostimulants i.e., protein hydrolysate (PH), seaweed extracts (SW), and their combination PH + SW, compared to the control non-stressed plants **(C)**. In the panel shows **(A)** photosynthetic performance expressed as quantum yield of the photosystem II (Phi2), ratio of incoming light that is lost *via* non-regulated processes (PhiNO) and non-photochemical quenching (PhiNPQ). In **(B)** relative chlorophyll content. In **(C)** % of relative water content (RWC) in tomato leaves. In **(D)** % of membrane stability index. Error bars indicate standard errors (*n* = 3). Different letters indicate significant differences according to Tukey’s multiple-range test (*p* = 0.05).

The agronomical and physiological results of tomato plants treated with protein hydrolysate (PH), seaweed extract (SW), and their combination PH + SW, were assessed to investigate their potential salinity stress mitigation (**Figures 1**, **2**).

For this purpose, at the end of the experimental period (18 DAT), different growing parameters were measured, including shoot and root fresh weight (FW; **Figure 1A**) and dry weight (**Figure 1B**), and shoot/root ratio (**Figure 1C**), together with representative images of the morphological differences (**Figure 1D**). Specifically, the FW and DW measurements pointed out, on average, a 57% biomass reduction (p< 0.05) under salinity stress compared to the control. The application of biostimulants results in increased stress tolerance and reduced biomass loss. In particular, the shoot biomass tended to increase in response to PH biostimulant application, while the root biomass for SW employment. This tendency was statistically significant (p< 0.05) compared to salinity-stressed plants. The combined application of PH and SW was found to be the worst in stress-mitigation potential at the morphological level, reporting no significant differences compared to salinity-stressed plants. Shoot/root ratio parameters highlighted the different mechanism of action driven by the two biostimulants. Indeed, PH application reported the highest and most significant values of shoot/root ratio compared to SW, indicating a preferentially shoot development driven by PH.

The photosynthetic performance was measured in terms of quantum yield of the photosystem II (Phi2), ratio of incoming light that is lost *via* non-regulated processes (PhiNO) and as non-photochemical quenching (PhiNPQ) (**Figure 2A**). In detail, salinity stress reduced the Phi2 and increased the PhiNPQ compared to the control. Interestingly, the decrease of photosystem II performance caused by salinity stress was reverted to the control condition after either PH or SW treatments. At the same time, the PH + SW application reported a distinctive behaviour, characterised by the lower performance of Phi2 and higher PhiNPQ values. Furthermore, PH and SW positively modulated the relative chlorophyll content in tomato leaves under salinity stress (**Figure 2B**).

Overall, salinity stress significantly increased the leaf relative water content (RWC; **Figure 2C**) and decreased membrane stability index (MSI; **Figure 2D**). However, the treatment with PH resulted in the only one capable of recovering MSI comparable to control plants.

### 3.2 Biostimulants differentially modulated tomato leaf mineral composition and organic acids accumulation

To gain insight into the mineral composition of leaves samples after biostimulant treatments, the main macronutrients, i.e., ${\text{NO}}_{3}^{-}$ , ${\text{PO}}_{4}^{3-}$ , K+, ${\text{SO}}_{4}^{2-}$ , Ca^2+^, Mg^2+^, Na^+^, and Cl^–^ have been quantified and reported in **Table 1**. Salinity stress modulated mineral accumulation in tomato leaves. In particular, ${\text{NO}}_{3}^{-}$ , K^+^, , and Ca^2+^ minerals were decreased under salinity stress, even though only ${\text{SO}}_{4}^{2-}$ was statistically significant compared to control. Accordingly, tomato plants grown in the presence of salinity stress (150mM NaCl) showed an intensive and significant accumulation of Na^+^ (7.27 g/kg DW) and Cl^–^ (85.59 g/kg DW) ions, compared to the control samples as 1.35 g/kg DW and 12.14 g/kg DW, respectively.

**Table 1:** Total minerals concentration of tomato leaves grown under salinity stress (S) and treated with biostimulants i.e., protein hydrolysate (PH), seaweed extracts (SW), and their combination PH + SW, compared to the control non-stressed plants (C).

	${\text{NO}}_{3}^{-}$	$\text{P}{\text{O}}_{4}^{3-}$	K^+^	$\text{S}{\text{O}}_{4}^{2-}$	Ca^2+^	Mg^2+^	Na^+^	Cl^–^
**g/Kg dry weight**								
**C**	0.99 ± 0.77 ^a^	3.33 ± 0.37	39.97 ± 1.19 ^b,c^	6.55 ± 0.3 ^a^	6.43 ± 1.72 ^a,b^	5.57 ± 0.77	1.35 ± 0.51 ^d^	12.14 ± 3.07 ^c^
**S**	0.6 ± 0.09 ^a,b^	3.14 ± 0.18	34.7 ± 2.71 ^c^	4.8 ± 0.41 ^b^	5.52 ± 0.7 ^b^	6.45 ± 0.83	7.27 ± 13.29 ^c^	85.59 ± 15.18 ^a^
**S + PH**	0.29 ± 0.12 ^b^	3.22 ± 0.35	43.35 ± 0.86 ^a,b^	5.95 ± 0.56 ^a^	7.42 ± 0.94 ^a^	5.6 ± 0.67	17.8 ± 14.82 ^b^	54.68 ± 6.98 ^b^
**S + SW**	0.41 ± 0.14 ^b^	3.07 ± 0.11	40.38 ± 8.6 ^b,c^	5.9 ± 0.78 ^a^	7.72 ± 0.88 ^a^	5.45 ± 0.53	12.94 ± 15.87 ^b^	51.07 ± 9.08 ^b^
**S + PH + SW**	0.73 ± 0.29 ^a,b^	3.21 ± 0.26	49.14 ± 5.86 ^a^	4.6 ± 1.18 ^b^	7.51 ± 0.56 ^a^	5.45 ± 0.7	25.15 ± 13.84 ^a^	74.92 ± 16.23 ^a^

All data are expressed as the mean ± standard error, n = 5, g/Kg dry weight. Different letters within each column indicate significant differences according to Tukey’s multiple-range test (p = 0.05).

Plants treated with biostimulants reported a clear modulation of mineral compositions. Indeed, both foliar sprayed PH and SW generated an accumulation of K^+^, ${\text{SO}}_{4}^{2-}$ and Ca^2+^ at a level equal to or above compared to salinity stressed plants. The combined application of PH and SW produced a diverse mineral accumulation profile, suggesting a different stress response mechanism. Particularly, the PH + SW application generated a clear and significant accumulation of K^+^. Moreover, a strong accumulation of Na^+^ and Cl^–^ was observed after PH + SW treatment. Interestingly, the application of biostimulants, both alone and in combination, produced a relevant accumulation of Na^+^, increasing by 2.44, 1.77, and 3.43 folds compared to that found in salinity stress only. This effect was not found in the case of Cl^–^ ion, which reported down accumulated under PH (54.68 g/kg DW) and SW (51.07 g/kg DW), and non-difference in the case of PH + SW treatment.

The effects of salinity stress on organic acids composition and content in tomato leaves were determined in terms of malate, oxalate, citrate and isocitrate quantification (**Table 2**). The malate and oxalate were reported a decreasing trend under salinity stress compared to the control, however, only malate variation was statistically significant. Instead, the behaviour of citrate and isocitrate showed an increasing trend, under salinity stress, although only citrate was statistically significant. The treatment with plant biostimulants modulated their accumulation considerably. Indeed, significant down-modulation of citrate and isocitrate was observed after PH and SW treatments on plants affected by salinity stress compared to the control.

**Table 2:** Total organic acids concentration of tomato leaves grown under salinity stress (S) and treated with biostimulants i.e., protein hydrolysate (PH), seaweed extracts (SW), and their combination PH + SW, compared to the control non-stressed plants (C).

	Malate	Oxalate	Citrate	Isocitrate
g/Kg dry weight				
**C**	28.24 ± 1.97 ^a^	1.82 ± 0.37 ^a^	10.94 ± 4.22 ^b^	0.5 ± 0.22 ^a,b^
**S**	20.92 ± 10.74 ^b^	1.51 ± 0.17 ^a,b^	16.46 ± 5.35 ^a^	0.61 ± 0.2 ^a^
**S + HP**	14.36 ± 1.05 ^b^	1.26 ± 0.14 ^b^	9.3 ± 2.46 ^b^	0.38 ± 0.09 ^b^
**S + SW**	17.15 ± 1.17 ^b^	1.36 ± 0.15 ^b^	9.35 ± 1.42 ^b^	0.36 ± 0.1 ^b^
**S + HP + SW**	15.18 ± 2.22 ^b^	1.17 ± 0.28 ^b^	12.24 ± 3.47 ^a,b^	0.38 ± 0.19 ^b^

All data are expressed as the mean ± standard error, n = 5, g/Kg dry weight. Different letters within each column indicate significant differences according to Tukey’s multiple-range test (p = 0.05).

### 3.3 Biostimulants application actively modulated essential metabolic pathways involved in stress response

The different metabolic responses induced by biostimulant application on tomato plants subjected to salinity stress were investigated using an untargeted metabolomics approach *via* UHPLC-ESI/QTOF-MS. The untargeted profiling allowed us to putatively annotate more than 3300 metabolites. Their comprehensive list is reported in the supplementary materials (**Table S1**), including compounds name, pathways, ontology classification, peaks abundances, retention time and masses of each metabolite.

The unsupervised hierarchical cluster analysis (HCA) was performed on the entire dataset to point out similarities/dissimilarities among treatments based on detected metabolites (**Figure S1**) and resulted in two main clusters. The first one showed clear metabolic profile similarities among plants treated with PH and the control, representing a unique subcluster, followed by PH + SW treatment (second subcluster). The second cluster was characterized by metabolites modulated by SW application and salinity stress. Although the application of SW reported distinctive morpho-physiological characteristics, the leaves’ metabolic profiles resulted in no separation between stressed plants, suggesting a different mechanism of action involved.

**Figure 3:**
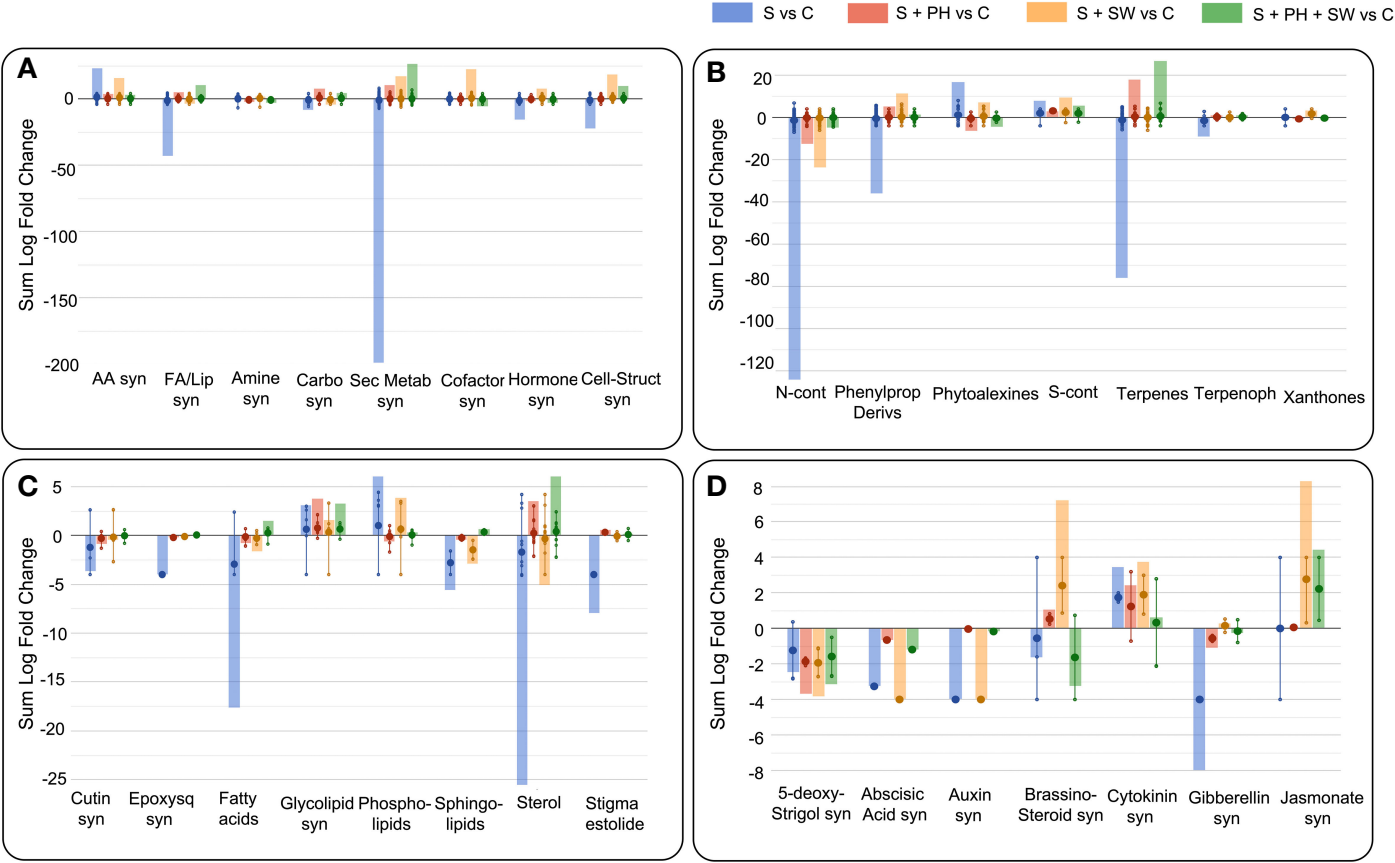
Pathway analysis of tomato leaves affected by salinity stress **(S)** and treated with biostimulants i.e., protein hydrolysate (PH), seaweed extracts (SW), and their combination PH + SW, compared to the control non-stressed plants **(C)**. The metabolites used to carry out the pathways analysis are those that have passed ANOVA (p-value< 0.05) and fold change (FC ≥ 2.5) analysis. Differential metabolites were interpreted in terms of biosynthesis pathways **(A)**, secondary metabolite **(B)**, lipids and fatty acids **(C)** and hormone biosynthesis (**D**). Error bars indicate the standard deviation of logFC values of all compounds belonging to the class. The larger dot indicates the median value, while the smaller dot indicates the actual value of each individual compound belonging to the class. The bars indicate the algebraic sum of the logFC values of the individual compounds belonging to the same class. Abbreviation = syn: biosynthesis; AA: amino acid; FA/Lip: fatty acid and lipid; Carbo: carbohydrate; Sec Metab: secondary metabolites; Cell-struct: cell-structure; N-cont: nitrogen-containing; Phenylprop: phenylpropanoids; S-cont: sulphur-containing; Terpenoph: terpenophenolics; Epoxysq: epoxysqualene.

The Volcano Plot analysis was used to investigate the mechanism of action of the biostimulants on tomato under salinity, combining ANOVA statistical analysis (p< 0.05) and fold change analysis (FC, threshold of 2.5). Overall, 609 differential metabolites were identified, and their complete list is provided in the Supplementary material (**Supplementary Table S2**). The graphical representation of the resulting biosynthetic pathways is reported in **Figure 3**, including biosynthetic pathways (**Figure 3A**), secondary metabolites biosynthesis (**Figure 3B**), lipids and fatty acids biosynthesis (**Figure 3C**), and hormones biosynthesis (**Figure 3D**), as well as the summary pathway table in **Supplementary Table S3**.

The main biosynthetic pathways modulated by salinity stress in tomato leaves, the biosynthesis of the secondary metabolites reported the highest down-modulation, followed by fatty acids and lipids and phytohormones biosynthesis. Instead, a clear accumulation was observed for amino acid biosynthesis (**Figure 3A**). Focusing on the pathways most affected by salinity stress, such as secondary metabolism, the down-modulated compounds belong to the macroscopic classes of nitrogen-containing, phenylpropanoids, terpenes, and terpenophenolics compounds. In contrast, classes of compounds belonging to phytoalexins and sulphur-containing, were up-modulated (**Figure 3B**). Considering fatty acids and lipids biosynthesis, the salinity stress negatively affected the biosynthetic pathways of sterols, fatty acids, sphingolipids, and cutin biosynthesis (**Figure 3C**).

Interestingly, foliar spray biostimulants application reported an effective modulation on those pathways damaged by salinity stress. Indeed, secondary metabolites following biostimulant application were up-modulated, and this trend was in common with fatty acids and lipids biosynthesis. Biostimulant treatments mitigated or even reverted both the biosynthesis of secondary metabolites such as nitrogen-containing, phenylpropanoids and terpenes compounds (**Figure 3B**), and the biosynthesis of fatty acids and lipids including sterols, fatty acids, and sphingolipids (**Figure 3C**).

Between the different biostimulants, in many cases, SW was found less effective in mitigating salinity stress-damaged pathways compared to PH and PH + SW. This was evident for secondary metabolites (nitrogen-containing, phytoalexins, and terpenes) and in lipids (fatty acids, phospholipids, sphingolipids, and sterols) biosynthesis. Indeed, PH reported a distinctive modulation pattern and their combination with SW. In particular, the combination of PH + SW showed an opposite modulation trend compared to stressed plants, suggesting a different stress response mechanism.

Phytohormones’ biosynthetic pathways were also affected by salinity stress modulation, as well as the application of biostimulants (**Figure 3D**). Indeed, metabolites involved in the biosynthesis or degradation of 5-deoxy-stringol, abscisic acid (ABA), auxin, brassinosteroid, cytokinin, gibberellin, and jasmonate were reported to be highly modulated. Specifically, salinity stress firmly modulated gibberellin, cytokinin, auxin, ABA, and 5-deoxy-stringol biosynthesis. Instead, SW treatment strongly modulated brassinosteroid and jasmonate biosynthesis, comparted to PH treated one. The combination of PH + SW reported a distinctive modulation profile compared to their single components. However, given the low half-life of phytohormones, metabolites involved in both biosynthesis and degradation were considered to have a positive modulation.

## 4 Discussion

Salinity stress is a major environmental stress that affects crops’ physiological, morphological, and biochemical processes, limiting their growth and productivity. According to literature, tomato plants grown under a high salt concentration (150 mM) were highly inhibited in the growing potential, reporting more than 50% of biomasses reduction, both considering FW and DW parameters. This effect was also observed by Tanveer et al. (2020). This imminent morphological effect was due to hyperosmolarity of the soil solution, which is translated into the plant system through two main phases: (1) a rapid osmotic stress response, which is independent of salt accumulation in the shoot, that causes plant growth inhibition (Almeida et al., 2017), and (2) plant adaptions to salinity stress by activation of different intracellular osmotic stress tolerance mechanism (Zhao et al., 2021). The resilience mechanism includes Na^+^ or Cl^−^ exclusion, tissue tolerance, a modulation of signal molecules, stomatal closure and the maintenance of the water status (Munns and Tester, 2008; Pardo, 2010). The application of biostimulants on tomato plants under salinity has reported a mitigating effect in terms of growth parameters, both on root and shoot organ parts. As known from the literature on salinity stress, in most cases Na^+^ and not Cl^-^ is considered the ion that first reaches a toxic concentration in root of rice seedlings (Chi Lin and Huei Kao, 2001; Tsai et al., 2004). However, for several species, Cl^−^ is represented as the most toxic ion because Na^+^ is usually withheld in roots and stems and only a small part reaches the leaves (Almeida et al., 2017). Conversely, Cl^−^ passes freely to the lamina, becoming the most significant toxic component under salt stress (Storey and Walker, 1998). According to our results, salinity stress significantly modulated Cl^−^ accumulation in saline-stressed plants (7 folds), whereas the employment of PH and SW reduced this value to about 4 folds. The combined PH + SW reported non-significant modulation in this Cl^−^ accumulation compared to stressed plants.

The localization of Na^+^ on the roots and stems occurs mainly when the concentration of Na^+^ in the soil solution increases and promotes in passive Na^+^ transport by a family of Non-Selective Cation Channels (NSCCs family) within root cells. Na^+^ taken up by the roots are then transported to shoots *via* xylem by bulk flow (Almeida et al., 2017). This process is driven by movement of water from the root through the plant to the surrounding atmosphere during transpiration. However, Na^+^ is also the primary toxic ion as it interferes with the uptake of K^+^ that is involved in the stomatal regulation (Tavakkoli et al., 2010). In this latter case, the water transpiration is limited by stomata closure through the action of K^+^ to avoid water loss, thus leading to the segregation of Na^+^ mainly in roots and stems compartment (Tavakkoli et al., 2010). Moreover, the Na^+^ efflux process is considered to be an unfavourable action as it occurs through the action of Na^+^/H^+^ antiporters by the consumption of ATP and PPi (Zhao et al., 2021). Another mechanism that limits Na^+^ accumulation in leaves is the Na^+^ retrieval mechanism. Plants can reabsorb Na^+^ from the xylem to the root cells as a mechanism to prevent large Na^+^ accumulation in aboveground tissues (Maathuis et al., 2014). This finding was confirmand in *Arabidopsis*, where the disruption of HKT1 gene (Na^+^ transporter) leads to hypersensitivity to salinity in mutant lines, with increased Na^+^ in leaves. The knockout lines showed a higher amount of Na^+^ in the shoots but a lower level of K^+^ (Møller et al., 2009).

The application of biostimulants on tomato plants under salinity stress reported an increase of Na^+^ accumulation in leaves, compared to the control. However, the beneficial effect of PH and SW biostimulants application were observed in terms of Phi2, PhiNPQ, and chlorophyll content, suggesting a different mechanism of action than compartmentalization and/or exclusion of toxic ions. In fact, plant responses to salt stress is also manifested by production of secondary signaling molecules such as ROS, contributing to the deleterious effects on plants (Mittova et al., 2004; Bai et al., 2018). Application of PH and SW biostimulants could enhance the biosynthesis of antioxidant agents to cope with salinity-driven oxidative stress and increase the amount of Na^+^ ion tolerance. Indeed, the application of PH and SW clearly up-modulated powerful antioxidant compounds like phenolics and flavonoids, reported to have also a high antioxidant activity under salinity stress in *Salvia mirzayanii* (Valifard et al., 2014), *Carthamus tinctorius* L. (Golkar and Taghizadeh, 2018), *Phaseolus vulgaris* L. (Semida and Rady, 2014).

Salinity stress severely affects the photosynthesis apparatus caused by the reduction of plant water potential and chlorophyll biosynthesis. Giordano et al. (2021) reported the involvement of Cl^−^ in the biosynthesis of chlorophyll, which higher concentration interferes with its production. Interestingly, the negative correlation between leaf Cl^−^ content and chlorophyll production and photosynthetic performance in our plants, confirming the toxicity of Cl^-^ accumulation on the photosynthetic system of leaves. Accordingly, PH and SW showed the best ones in mitigating salinity stress in the photosynthesis activity and chlorophyll concentration.

Usually, the effect of salinity stress is superimposed on those caused by dehydration. Indeed, salinity stress is often associated with water deficit, as the salt dissolved in the soil reduces water availability and water uptake by the roots (Munns and Tester, 2008). RWC is a parameter that provides the degree of hydration of plant tissue and is considered a very important parameter for determining the maximum water contained in the plant Teulat et al. (1997). The tomato plants under salinity stress reported a value of RWC greater than those observed in the control plants. Although the RWC parameter has been reported by Suriya-arunruj et al. (2004) as an effective method to evaluate salt stress tolerance, where a high RWC value has been correlated with high salt stress tolerance, this observation was not in agreement with our data. However, the high RWC content in our salinity-stressed plants could be explained by the fact that water retention is a primary defense system adopted by plants under water shortage caused by osmotic effect (Zhu, 2002; Zhao et al., 2021). Indeed, sustained transpiration without sufficient water supply leads to loss of leaf turgor, decrease of photosynthetic activity, and growth capacity (Acosta-Motos et al., 2017). Similarly, no mitigating effect was reported after biostimulants application. Higher RWC values could be attributed to the leaf stomatal closure under salinity stress. In fact, in normal growth conditions (i.e., well-watered plants) leaf stomata are fully open during daylight periods, when light stimulates stomatal opening *via* blue light-specific and photosynthetic-active radiation-dependent pathways, to maximize the assimilation of CO_2_ and ensure an optimal photosynthesis rate (Roelfsema and Hedrich, 2005). However, under stress conditions, stomata were closed to reduce water loss, with a consequent reduction in photosynthesis (Giménez et al., 2013; Giordano et al., 2021). Accordingly, salt-stressed tomato plants also reported a reduced value of Phi2 and increased value of PhiNPQ, as well as low chlorophyll content, compared to the control. Therefore, the high RWC value observed in the stressed samples is purely attributed to a saline stress response status activated by the tomato plant, resulting in the closure of the stomata and the following accumulation of water in the leaves. The stoma closure process is directly regulated by K^+^ efflux through depolarisation-activated K^+^ channels in guard cells (Demidchik, 2014). Due to the nature of the K^+^ channels, it also requires the movement of counterions to balance the plasma membrane potential, such as Cl^−^, ${\text{PO}}_{4}^{3-}$ , NO^3−^, citrate, and malate (Demidchik, 2014; Khan et al., 2020). Accordingly, salinity stress-affected tomato plants reported a low trend accumulation of K^+^ and higher accumulation of potential counterions Cl^−^and citrate in their leaves. However, biostimulants treated plants reported a different profile of mineral content in leaves, suggesting a different stress response mechanism, despite the similar RWC values reported. Indeed, contrary to what was observed for the stressed samples, the application of PH reported an up-accumulation of K^+^ and a milder decreased content of Cl^−^ and citrate, compared to stressed plants. In this sense, it is interesting the role of Ca^2+^, which was observed to inhibit the efflux of K^+^ under salinity stress (Davies and Newman, 2006). Accordingly, the Ca^2+^ level in plants treated with biostimulants were higher than those detected in salt-stressed leaves. Moreover, the beneficial effect of up-accumulated Ca^2+^ in treated plants is also correlated to the membrane stability index, as confirmed by several authors (Tuna et al., 2007; Khursheda et al., 2015; Tanveer et al., 2020). Specifically, salinity stress affected MSI of tomato plants severally, compared to non-stressed ones. However, the application of PH reported a clear mitigation capability on membrane integrity. This beneficial effect could be attributed to biostimulants’ ability to increase Ca^2+^ coupled to enhancement of sterols biosynthesis, both involved in the regulation of membrane stability and permeability (Tuna et al., 2007; Guo et al., 2019).

The Ca^2+^ and K^+^ could be attributed to the phytohormones regulation. Indeed, salt-stressed plants reported an overall down modulation of hormones biosynthesis, except for cytokinin and jasmonate. The application of PH and SW up-modulated the metabolites involved in the biosynthesis of brassinolide (BR), while SW and PH + SW application modulated those in the biosynthesis of jasmonic acid (JA), and finally all three biostimulants reported a modulatory capacity of gibberellin biosynthesis (GB). These phytohormones were reported to have a positive relationship to salinity tolerance response. The BR accumulation is directly related to the enhancement of plant antioxidant activity by implementing the activity of antioxidant enzymes and accumulating antioxidant compounds such as tocopherol, glutathione and polyphenols (El-Mashad and Mohamed, 2012). According to the literature, the application with SW reported an up modulation of BR and the following accumulation of antioxidant compounds, as well as the up modulation of phenylpropanoid biosynthesis pathways (Goñi et al., 2018; Deolu-Ajayi et al., 2022). Similarly, the up-regulation of JA is related to attenuating salinity stresses by accumulation of osmolytes to limit the uptake of Na^+^ and Cl^−^ and non-enzymatic antioxidants such as flavonoids (Yastreb et al., 2016).

As we observed, Ca^2+^ ions mediate several mechanisms of the salinity stress response due to their flexibility in exhibiting different coordination numbers and forming complexes with proteins, membranes, and organic acids (Kudla et al., 2010). Indeed, the Ca^2+^ ion is the most important secondary messenger in plants. A potential alteration of the Ca^2+^ signalling cascade could regulate a wide range of physiological processes, including gene expression, protein activities, and biosynthetic pathways (*i.e.*, secondary metabolites, fatty acids and lipids, phytohormones, and other pathways) (Kudla et al., 2010; Roy et al., 2014). In regulating lipids biosynthesis, Ca^2+^ plays an essential role in processes that preserve the structural and functional integrity of plant membranes, stabilize cell wall structures, regulate ion transport and selectivity, and control ion exchange (Tuna et al., 2007). As well known, MSI is closely related to the composition of membrane lipids and strong changes in lipid composition induced by salinity stress could severally affect the membrane fluidity, permeability, and electrolyte leakage (Tuna et al., 2007; López-Pérez et al., 2009). In our samples, a strong down modulation of membrane lipid composition was observed in tomato plants affected by salt stress. The main changes involve the composition of sterols, sphingolipids, phospholipids, and fatty acids. Salinity stress strongly down-modulated the sterols and fatty acids synthesis, whereas biostimulants application reverted this negative effect. Particularly for phospholipids, we found a relevant up-accumulated under salinity stress and this observation was also supported by Salama and Mansour (2015). Interestingly, phospholipids were down-modulated after PH and slightly PH + SW applications. Concerning phytosterols, they were reported to be heavily down-modulated by salt stress. However, both PH and PH + SW applications resulted in an up-modulation of these lipids. Sterols are important structural components being constitutively down accumulated in plant membranes affected by salt stress (Guo et al., 2019). Salama and Mansour (2015) reported that maintaining a constant level of sterols in the membrane would be essential for plant salt tolerance. PH and PH + SW treatments stimulated the synthesis of planar sterols such as campesterol, brassicasterol, and 7-dehydrocholesterol, which are thought to regulate membrane fluidity and permeability in plant membranes by restricting the mobility of fatty acyl chains (Guo et al., 2019). The same result has been achieved for fatty acids composition, with salt stress strongly down modulated their biosynthesis despite a recovery could be observed after biostimulant application. Specifically, PH and PH + SW treated plants reported an improvement in saturated fatty acids *i.e.*, palmitoyl-CoA and lauroyl-CoA, which improve liquid-order phases that are directly related to membrane fluidity (Guo et al., 2019). The MSI reported from agronomical results were according to PH-treated tomato, but not in the case of PH + SW-treated plants. The reason could be due to the unsaturated fatty acid composition, which generates liquid-disordered phases and, consequently membrane instability (Guo et al., 2019). Unsaturated lipids were up accumulated under SW and PH + SW treatments, corroborating the morpho-physiological data reported from our results. In this case, we can state that the beneficial effect of PH treatment is strictly correlated with their ability to modify the composition of membrane lipids and change the ratio between saturated and unsaturated fatty acids. This effect is not evident in PH + SW treatment and is null in the case of SW application. The emerging evidence of the SW-driven mechanism of action may lie in the ability of SW to stimulate secondary metabolism, in particular in the production of phenylpropanoids and their antioxidant capacity (Deolu-Ajayi et al., 2022). Phenylpropanoids as well as nitrogen-containing and terpenes, were the secondary metabolites mostly affected by salinity stress in tomato plants. The biosynthesis of polyphenols is usually decreased in salt-stressed plants, as confirmed by several authors (Eryılmaz, 2006; Ben Dkhil and Denden, 2012; Yan et al., 2014; Wang et al., 2016; Bistgani et al., 2019). However, the biostimulants were able to up modulate their production, particularly under SW treatment. Plant phenolic compounds are involved in key metabolic and physiological processes, including growth and development, synthesis of photosynthetic pigment, and scavenging of harmful ROS (Golkar and Taghizadeh, 2018; Bistgani et al., 2019; Sharma et al., 2019). The scavenging of ROS is the most important beneficial property of polyphenols because the accumulation of salt-induced ROS is closely related to the activation of specific ROS-promoted signaling pathways, such as processes involved in the damage of photosynthetic systems, limitation of CO_2_ fixation, as well as peroxidation and destabilization of cellular membranes (Mittova et al., 2004). Moreover, generated ROS could also damage vital molecules such as nucleic acids, proteins, carbohydrates, and lipids (Sharma et al., 2019). Another mechanism of salinity stress-mitigation adopted by biostimulants was the modulation of N-containing compounds and terpenes. N-containing compounds are compounds characterized by a nitrogen group in their structure, such as glucosinolates, their hydrolysis products, and alkaloids. The relevant function of these classes of compounds in abiotic stress mitigation has been described by Del Carmen Martínez-Ballesta et al. (2013). The author also suggested a correlation between the increase of glucosinolates production and the synthesis of osmoprotective compounds e.g., proline (Matysik et al., 2002), glycine betaine (Mäkelä et al., 2000), and sugars (Saxena et al., 2013). Osmolytes are mainly involved in protecting the functions of cell structures and maintaining osmotic balance under salinity stress (Hare et al., 1999; Ghosh et al., 2021). As aforementioned, tomato plants treated with biostimulants showed an up-modulation of the precursors for the synthesis of proline (PH treatment) and betaine (SW treatment), as well as in the biosynthesis of carbohydrates (PH + SW treatment). Concerning the class of terpenes, we found a strong modulation of carotenoids and precursor for the biosynthesis of phytosterols, driven mainly by PH-based treatment, in accordance with the chlorophyll content and MSI results previously reported.

## 5 Conclusion

The search for sustainable approaches able to sustain crop productivity under unfavorable conditions has paved the way towards the use of plant biostimulants in agriculture. However, unraveling the mechanisms involved in biostimulants beneficial effects becomes of pivotal importance to identify tailored applications in line with the different agricultural scenarios worldwide. Our results evidenced a beneficial contribution of the tested biostimulants in terms of salinity stress mitigation. In agreement with previous reports on biostimulants, a broad metabolomic reprogramming could be observed following the application of biostimulants, with secondary metabolites, membrane lipids and phytosterols, as well as hormones showing the most extensive modulation.

Interestingly, distinct effects could be observed when the protein hydrolysate or the seaweed extract were applied, either alone or in combination, corroborating the differences observed at metabolomics level. On one side, this supports the need for understanding the mode of action, to properly design biostimulants-based agricultural solutions. On the other side, this opens the possibility towards an integrated strategy that uses complementary biostimulants, provided that synergistic effects are demonstrated. Starting from our results, a comprehensive evaluation that goes beyond plant science and includes also economic and environmental sustainability issues is recommended.

## Data availability statement

The original contributions presented in this study are included in the article/**Supplementary Material**. Further inquiries can be directed to the corresponding author.

## Author contributions

LZ, GF, YR, SP, and LL: Conceptualization and project administration. LZ, GF, YR, SP, and LL: Methodology, validation, formal analysis, investigation, writing—original draft preparation. SDP and LL: Writing—review and editing. LZ: Software and data curation. GF, SP, YR and LL: Resources. LL: Visualization and supervision. All authors contributed to the article and approved the submitted version.
