# Supplementary material for: The differential modulation of secondary metabolism induced by a protein hydrolysate and a seaweed extract in tomato plants under salinity
Source: Front Plant Sci. 2023 Jan 16;13:1072782. doi: 10.3389/fpls.2022.1072782 (PMC9884811; doi:10.3389/fpls.2022.1072782)
Supplement: Supplementary file 1 [file DataSheet_1.docx]

Supplementary Material

# Supplementary Figures


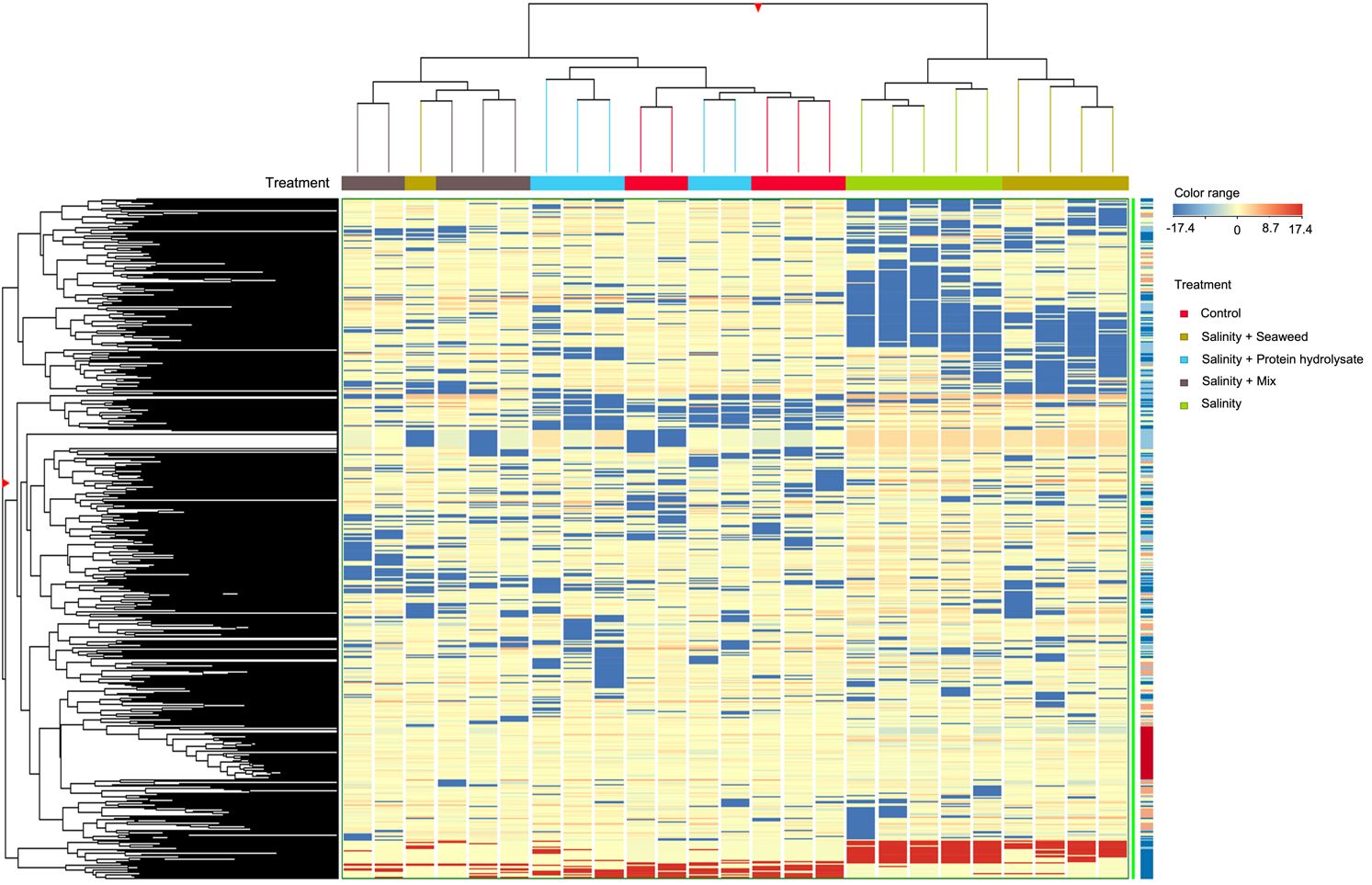


**Supplementary Figure 1.** Unsupervised hierarchical cluster analysis (HCA) was created using metabolite profiles of tomato leaves treated with biostimulants *i.e.,* protein hydrolysate, seaweed extracts, and their combination (Mix), affected by salinity stress.

# Supplementary Tables

**Supplementary table S1.** Comprehensive dataset of tomato leaves affected by salinity stress and treated with biostimulants i.e., protein hydrolysate, seaweed extracts, and their combination, compared to the control non-stressed plants, obtained from UHPLC-QTOF-MS untargeted metabolomics analysis.

**Supplementary table S2.** Volcano analysis (p-value < 0.05 and FC ≥ 2.5) of tomato leaves affected by salinity stress and treated with biostimulants i.e., protein hydrolysate, seaweed extracts, and their combination, compared to the control non-stressed plants.

**Supplementary table S3.** The metabolites used to carry out the pathways analysis of biosynthesis, secondary metabolites biosynthesis, lipids and fatty acids biosynthesis, and hormones biosynthesis pathways.
